# Role of the Slug Transcription Factor in Chemically-Induced Skin Cancer

**DOI:** 10.3390/jcm5020021

**Published:** 2016-02-03

**Authors:** Kristine von Maltzan, Yafan Li, Joyce E. Rundhaug, Laurie G. Hudson, Susan M. Fischer, Donna F. Kusewitt

**Affiliations:** 1 Department of Epigenetics and Molecular Carcinogenesis, University of Texas M.D. Anderson Cancer Center, P.O. Box 389, Smithville, TX 78957, USA; kvmaltzan@gmail.com (K.V.M.); Yafan.Li@Lubrizol.com (J.E.R.); jerundhaug@austin.rr.com (S.M.F.); 2 Program in Toxicology and Pharmacology, College of Pharmacy, University of New Mexico Health Sciences Center, MSC 09 5360, 1 University of New Mexico, Albuquerque, NM 87131, USA; Yafan.Li@Lubrizol.com (Y.L.); LHudson@salud.unm.edu (L.G.H.)

**Keywords:** slug, snail, epithelial-mesenchymal transition, skin carcinogenesis

## Abstract

The Slug transcription factor plays an important role in ultraviolet radiation (UVR)-induced skin carcinogenesis, particularly in the epithelial-mesenchymal transition (EMT) occurring during tumor progression. In the present studies, we investigated the role of Slug in two-stage chemical skin carcinogenesis. Slug and the related transcription factor Snail were expressed at high levels in skin tumors induced by 7,12-dimethylbenz[α]anthracene application followed by 12-*O*-tetradecanoylphorbol-13-acetate (TPA) treatment. TPA-induced transient elevation of Slug and Snail proteins in normal mouse epidermis and studies in Slug transgenic mice indicated that Slug modulates TPA-induced epidermal hyperplasia and cutaneous inflammation. Although Snail family factors have been linked to inflammation via interactions with the cyclooxygenase-2 (COX-2) pathway, a pathway that also plays an important role in skin carcinogenesis, transient TPA induction of Slug and Snail appeared unrelated to COX-2 expression. In cultured human keratinocytes, TPA induced Snail mRNA expression while suppressing Slug expression, and this differential regulation was due specifically to activation of the TPA receptor. These studies show that Slug and Snail exhibit similar patterns of expression during both UVR and chemical skin carcinogenesis, that Slug and Snail can be differentially regulated under some conditions and that *in vitro* findings may not recapitulate *in vivo* results.

## 1. Introduction

Epithelial-mesenchymal transition (EMT) is characterized by loss of homotypic adhesion, acquisition of migratory capabilities and a switch from keratin to vimentin intermediate filament expression. EMT occurs prominently during development, particularly during gastrulation, neural crest cell emigration and fusion of midline head structures [1,2,3,4]. Slug (Snai2) and Snail (Snai1), members of the Snail family of zinc finger transcriptional repressors, play important roles in the EMT occurring during embryonic development [3,4]. In adult tissues, Snail family members modulate the EMT-like process of carcinoma progression [1,4,5,6]. By driving decreased adhesion and enhanced motility during carcinoma progression, Snail factors are critical determinants of tumor invasiveness and metastasis.

EMT takes place during ultraviolet radiation (UVR) and chemically-induced skin carcinogenesis in mice, as tumors progress from a squamous to a spindle morphology. UVR rapidly induces transient expression of Slug and Snail in keratinocytes [7], and expression of both Snail and Slug is elevated in UVR-induced skin tumors compared to normal epidermis [8]. However, patterns of Slug and Snail expression during skin carcinogenesis are not identical. Although Slug is more highly expressed in UVR-induced skin tumors with an epithelial *versus* spindle cell morphology, Snail expression is higher in spindle cell than in epithelial tumors. With chronic UVR exposure, Slug knockout mice develop a smaller skin tumor burden than wild-type mice, with fewer spindle cell carcinomas [8]. Although conversion from a squamous to spindle cell morphology does occur in the tumors of Slug knockout mice, the spindle cell tumors that develop have a more epithelial pattern of gene expression than spindle cell tumors in wild-type mice. Snail transgenic mice subjected to a two-stage chemical carcinogenesis protocol show enhanced skin tumor development; however, the tumors that develop in Snail transgenic mice are sebaceous carcinomas, rather than squamous cell carcinomas [9].

Slug and Snail also contribute to skin carcinogenesis by enhancing local inflammation. Transgenic mice that ectopically express Snail in the epidermis have increased numbers of inflammatory cells in the dermis, indicating a role for Snail in cutaneous inflammation [9]. Slug knockout mice are resistant to sunburn, developing a much less robust dermal inflammatory response than wild-type mice; decreased inflammation in Slug knockout mice appears to be due to the failure of the UVR-exposed keratinocytes to release proinflammatory mediators [10].

Prostaglandins generated by cyclooxygenase-2 (COX-2) are potent mediators of inflammation and proliferation and make substantial contributions to UVR and chemical skin carcinogenesis [11,12]. In UVR-exposed skin, as well as in non-small-cell lung and pancreatic cancer, Slug transcriptionally represses 15-hydroxyprostaglandin dehydrogenase [3,9,13,14]. This enzyme catabolizes prostaglandins and reduces inflammation, thus acting as a tumor suppressor. It has also been reported that suppressing Snail expression in non-small-cell lung cancer cells decreases expression of COX-2 [14]. On the other hand, some studies indicate that COX-2 may modulate expression of Slug and Snail [15,16,17]. Thus, the interrelationship between COX-2 and Snail family expression remains to be clarified. It is clear, however, that COX-2 can stimulate EMT-like enhanced motility and decreased homotypic adhesion in a variety of cancer cell types [18,19,20].

In previous studies, we showed that UVR induces expression of Slug and Snail in the epidermis, that Slug is persistently overexpressed in UVR-induced squamous cell carcinomas and that Slug modulates UVR-induced cutaneous inflammation. In the present studies of chemically-induced two-stage skin carcinogenesis, we demonstrate that Slug and Snail are induced by the tumor promoter 12-*O*-tetradecanoylphorbol-13-acetate (TPA), that Slug and Snail are persistently overexpressed in 7,12-dimethylbenz[α]anthracene (DMBA)/TPA-induced squamous cell carcinomas and that Slug modulates TPA-induced epidermal hyperplasia and cutaneous inflammation. Thus, Slug appears to play a similar role during both UVR and chemically-induced skin carcinogenesis. In addition, our studies indicate that COX-2 does not appear to play a role in TPA induction of Slug and Snail and highlight the finding that *in vivo* and *in vitro* studies of Slug induction may yield different results.

## 2. Experimental Section

### 2.1. Animal Studies

Six 8-week-old females of the FVB strain (Harlan, Indianapolis, IN, USA) were employed for carcinogenesis studies and to examine the acute response to single doses of TPA. For carcinogenesis studies, the backs of the mice were shaved 2–3 days before application of 100 µg DMBA in 200 µL acetone. Starting 2 weeks later, 2.5 µg TPA (LC Laboratories, Woburn, MA, USA) in 200 µL acetone were applied twice weekly. Tumor-bearing mice were killed by CO_2_ inhalation followed by cervical dislocation 25–35 weeks after TPA treatment began. Portions of skin or tumors were snap frozen in liquid nitrogen and stored at −80 °C. Remaining skin or tumors were fixed in neutral-buffered formalin. For studies of the acute response of the skin to TPA, 2.5 µg TPA in 200 µL acetone or acetone only was applied once to the shaved backs of the mice. Mice were killed and skin collected at selected times after TPA application.

Previously-described transgenic mice with exogenous Slug expression driven by the keratin 5 promoter [21] and their wild-type littermates were used to examine TPA induction of epidermal proliferation and acute inflammation; mice were on a pure FVB background, and transgenic mice with only a single copy of the transgene were employed. TPA (2.5 µg in 200 µL acetone) or acetone only was applied once to the shaved backs of 6-month-old male and female mice; mice were killed, and skin was collected 24 h later.

To examine the effect of COX-2 inhibitors on the TPA response, FVB mice were fed diets containing powdered AIN-76 diet (Research Diets, New Brunswick, NJ, USA) containing 4 ppm indomethacin (Sigma, St. Louis, MO, USA) or 750 ppm celecoxib (LKT Labs, St. Paul, MN, USA) for one week; mice were then shaved, and TPA was applied as described above. For studies of PGE2 levels, previously-described Slug knockout mice [22] and wild-type 129 littermates were used; all mice were males, approximately 5 months of age. TPA (2.5 µg in 200 µL acetone) or acetone only was applied once to the shaved backs of the mice, and skin was collected and frozen 6 h after treatment.

Animal studies were performed in accordance with all applicable state and federal animal welfare regulations, using protocols approved by the MD Anderson Cancer Center Institutional Animal Care and Use Committee.

### 2.2. Histology and Image Analysis

Skin for H & E staining and immunohistochemistry was flattened on thin cardboard, fixed 24–48 h in neutral-buffered formalin and stored in 70% ethanol until dehydrated and embedded in paraffin using routine procedures. For immunohistochemistry, endogenous peroxidase activity was blocked by incubation with 3% H_2_O_2_; antigen retrieval was performed by microwaving the samples in 10 mM citrate buffer (pH 6.0); non-specific antibody binding was blocked with Biocare Blocking Reagent (Concord, CA, USA); and primary antibodies were added for overnight incubation at 4 °C. Antibodies included a rabbit monoclonal anti-Slug antibody (Cell Signaling, Danvers, MA, USA) diluted 1:50, a goat polyclonal anti-Snail antibody (R & D Systems, Minneapolis, MN, USA) diluted 1:250 and a rat monoclonal anti-Ly6G (BD Pharmingen, Franklin Lakes, NJ, USA) diluted 1:200. Detection was carried out by incubation for 30 min with Biocare Rabbit on Rodent HRP-Polymer, Biocare Goat HRP or biotinylated rabbit-anti-rat IgG (Vector Laboratories, Burlingame, CA, USA), as appropriate, followed by 3,3′-diaminobenzidine tetrahydrochloride (DAB). For Ly6G immunohistochemistry, the signal was amplified using a tyramide amplification kit (PerkinElmer, Waltham, MA, USA) according to the manufacturer’s instructions. Slides were then counterstained with hematoxylin, dehydrated and cover slipped.

Slides stained for Slug or Snail were digitized using an Aperio Scan Scope CS (Leica Biosystems, Buffalo Grove, IL, USA) and analyzed with the manufacturer’s GENIE software (Spectrum Version 10.2.2.2315). The standard DAB nuclear analysis program was modified to improve its ability to recognize immunopositive nuclei, and the modified analysis algorithm was applied to all slides. A minimum of 3 mm of representative epidermis was evaluated for each mouse. Epidermal thickness was determined on digitized images of H & E-stained slides at a total of 20 random sites for each mouse, using the Aperio ScanScope and GENIE morphometry program. The standard GENIE DAB nuclear analysis program successfully identified and quantified Ly6G-positive neutrophils. For each animal, at least 1 mm^2^ of dermis and subcutis was analyzed. Only regions of skin in telogen or early anagen were evaluated; hair follicles and sebaceous glands were excluded from analysis.

### 2.3. Cell Studies

Primary human keratinocytes (NHK) of neonatal origin were obtained from American Type Culture Collection (ATCC, Manassas, VA, USA) and used prior to passage 8. NHK were cultured in dermal cell basal medium (ATCC, Manassas, VA, USA) supplemented with a keratinocyte growth kit (ATCC, Manassas, VA, USA), 10 U/mL penicillin and 10 µg/mL streptomycin (Life Technologies, Carlsbad, CA, USA). Cells were seeded into 100 mm dishes at 2 × 10^6^ per dish and grown at 37 °C in 5% CO_2_ to confluence. Plates were then rinsed with PBS, and 10 mL serum-free medium (dermal cell basal medium supplemented with 1% bovine serum albumin) per plate were added for 24 h. TPA (30 ng/mL in acetone) or acetone was then added. The final acetone concentration in culture medium was 0.05%.

The SCC12F cell line, kindly provided to us by William A. Toscano, Jr. (University of Minnesota, Minneapolis, MN, USA), was derived from a well-differentiated human SCC arising on the face. SCC12F cells maintain a characteristic keratinocyte morphology, undergo density arrest and are non-tumorigenic in nude mice [23,24]. Cells were grown to confluence in a medium consisting of 50% Dulbecco’s Modified Eagle’s Medium, 50% Hams-F12 medium (Sigma, St. Louis, MO, USA), 0.5% penicillin and streptomycin, 1% l-glutamine and 5% fetal bovine serum (Gibco, Gaithersburg, MD, USA). Cells were maintained for 24 h in serum-free medium before TPA (6 ng/mL in 0.1% DMSO) or DMSO treatment. For some studies, cells were pretreated for 1 h with 6 μg/mL actinomycin D (Anaspec, San Jose, CA, USA) or for 15 min with the protein kinase C (PKC) inhibitor GF109203X (Calbiochem, La Jolla, CA, USA) at a concentration of 1 μM.

### 2.4. RNA Isolation and RT-PCR

Frozen tumors were ground to a fine powder in liquid nitrogen and lysed in 5 mL TriReagent (Molecular Research Center, Cincinnati, OH, USA). RNA was extracted per the manufacturer’s protocol and stored at −80 °C. To obtain RNA from mouse skin, frozen strips (~0.3 × 2 cm) of skin were placed, epidermis side down, on 500 µL of cold TRIzol (Life Technologies, Carlsbad, CA, USA) for 1 min; epidermis was then scraped from the surface. Epidermis was briefly homogenized in a total of 1 mL TRIzol. For isolation of total RNA from NHK cells, the cells were lysed with 1 mL cold TRIzol (Life Technologies, Carlsbad, CA, USA) per plate, and RNA isolation was performed as recommended by the supplier. Total RNA from SCC12F cells was isolated using the RNeasy RNA isolation kit following the manufacturer’s instructions (Qiagen, Valencia, CA, USA). One microgram of RNA was used for quantitative real-time RT-PCR (qRT-PCR) detection of Slug or Snail. Reverse transcription was performed using the High Capacity cDNA Reverse Transcription Kit from Life Technologies (Carlsbad, CA, USA) according to the instructions provided. For mouse samples, real-time qRT-PCR was performed using TaqMan gene expression assays (Life Technologies, Carlsbad, CA, USA) for Snail (Hs00195591_m1), Slug (Hs00161904_m1) and 18S RNA (Hs99999901_s1) according to the manufacturer’s specifications. For NHK, qRT-PCR was performed using TaqMan gene expression assays from Applied Biosystems (Life Technologies, Carlsbad, CA, USA): human Snail (HS00195591) and human Slug (HS00161904). Samples were run in duplicate, and results were normalized to 18S RNA (HS99999901_S1). Data were analyzed using the ΔΔC_t_ method (Schmittgen and Livak, 2008). Total RNA from SCC12F cells was isolated using the RNeasy RNA isolation kit following the manufacturer’s instructions (Qiagen, Hilden, Germany). One microgram of total RNA was reverse transcribed using the High Capacity cDNA Reverse Transcription kit (Applied Biosystems) according to the instructions from the manufacturer. Quantitative PCR was performed by monitoring in real time the increase in fluorescence of the SYBR Green dye on an ABI PRISM 7000 Sequence Detector System (Applied Biosystems, Foster City, CA, USA) according to the instructions from the manufacturer. Each real-time quantitative PCR assay was performed using duplicate samples and repeated for 3 independent samples. The expression level of 18S (Universal 18S Internal Standards kit, Ambion Inc., Austin, TX, USA) was measured in each sample using the same method for normalization. The primer sequences used included the following: Slug forward primer 5′-CCCTGAAGATGCATATTCGGAC-3′, Slug reverse primer 5′-CTTCTCCCCCGTGTGAGTTCTA-3′, Snail forward primer 5′-CGGAAGCCTAACTACAGCGA-3′ and Snail reverse primer 5′-GGACAGAGTCCCAGATGAGC-3′. The expression level of 18S (Universal 18S Internal Standards kit, Ambion, Austin, TX, USA) was measured in each sample using the same method for normalization.

### 2.5. Western Blotting

Whole cell extracts were prepared by lysing cells in RIPA buffer (50 mM Tris PH 7.4, 150 mM NaCl, 1 mM EDTA, 1 mM EGTA, 1 mM sodium orthovanadate, 1% NP40, 0.05% sodium dodecyl sulfate, 40 mM NaF, 10 mM sodium molybdate, 1 mM phenylmethylsulfonyl fluoride, 1% protein inhibitor) (Sigma, St. Louis, MO, USA) and centrifugation at 14,000 rpm for 10 min at 4 °C. For Western blotting, 25 µg of protein were loaded on 15% SDS-polyacrylamide gel, transferred to a nitrocellulose membrane and blocked in 5% powdered milk in TBST (50 mM Tris, pH 7.5, 150 mM NaCl, 0.01% Tween 20). The membrane was incubated with primary antibodies (anti-Slug or Snail at 1:5000 or anti-GAPDH at 1:2000) overnight at 4 °C in 5% powdered milk in TBST, washed extensively with TBST and incubated with a 1:5000 dilution of secondary antibody (Santa Cruz, Santa Cruz, CA, USA). Antibodies included a rabbit polyclonal antibody to Snail (Abcam, Cambridge, MA, USA), a rabbit polyclonal Slug antibody (a kind gift from Pascale Leroy at the University of California San Francisco) and a mouse monoclonal antibody to GAPDH (Millipore, Billerica, MA, USA). Proteins were visualized with the ECL detection kit (Amersham Pharmacia, Piscataway, NJ, USA).

### 2.6. Prostaglandin E2 Levels

Chipped frozen epidermis or whole skin was placed in 1 mL 0.05 M Tris-HCl, pH 7.4 containing 5 µg/mL indomethacin and homogenized. Proteins from the homogenate were precipitated with 4 volumes of 100% ethanol and centrifuged. The supernatant was evaporated under N_2_ gas and resuspended in 1 mL 0.05 M Tris-HCl, pH 7.4. After the addition of 2 volumes of 100% ethanol, samples were incubated on ice for 5 min; water was then added to a final concentration of 15% ethanol, and samples were acidified to ~pH 3 with HCl. Acidified samples were applied to C18 solid phase columns (Grace Davison Discovery Sciences, Deerfield, IL, USA); columns were washed with cold 15% ethanol and petroleum ether; and prostaglandins were eluted with cold methyl formate. Eluates were dried with N_2_, resuspended in enzyme immunoassay buffer, diluted as necessary and assayed for PGE2 by the enzyme immunoassay (Cayman Chemical, Ann Arbor, MI, USA) per the manufacturer’s protocol. Protein levels in the homogenate before precipitation were determined by the BCA method (Thermo Fisher Scientific, Rockford, IL, USA). PGE2 levels, assayed in duplicate at 2 or more dilutions in at least 2 separate assays, were determined as pg PGE2/mg protein. Epidermis from 4 TPA-treated wild-type and 4 TPA-treated Slug knockout mice was assayed. PGE2 levels were also determined in whole skin rather than epidermis of 2 untreated wild-type and 4 untreated Slug knockout mice.

### 2.7. Statistics

Differences between and among groups were analyzed using 1-way ANOVA followed by the Newman–Keuls multiple comparison test or the Bonferroni correction, the Kruskal–Wallis test followed by Dunn’s multiple comparison test, the Mann–Whitney test or the Student *t*-test, as appropriate. Analysis was carried out with Prism 5.0 software for Mac OS X (GraphPad Software, La Jolla, CA, USA) software.

## 3. Results and Discussion

### 3.1. Slug and Snail Are Expressed at Increased Levels in DMBA/TPA-Induced Tumors

**Figure 1:**
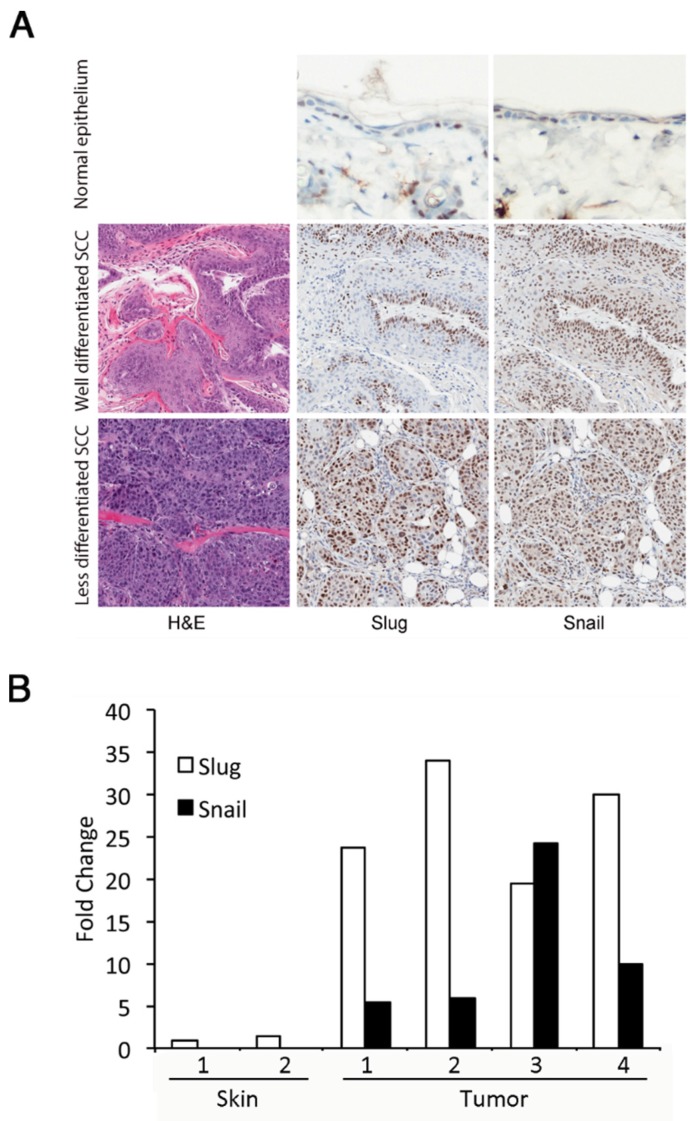
Slug and Snail are expressed at increased levels in 7,12-dimethylbenz[α]anthracene (DMBA)/12-*O*-tetradecanoylphorbol-13-acetate (TPA)-induced squamous cell carcinomas of the skin. (**A**) Immunohistochemistry for Slug and Snail was performed on normal untreated epidermis and on squamous cell carcinomas that arose in response to a standard DMBA/TPA two-stage carcinogenesis protocol. Representative normal epidermis and well-differentiated and more poorly differentiated tumors are shown. (**B**) mRNA from four of these squamous cell carcinomas was isolated, and the levels of Slug and Snail expression were determined by qRT-PCR. Expression in tumors was normalized to expression of Slug and Snail in the untreated epidermis (Skin Sample 1) of age- and strain-matched mice. Because Snail mRNA was undetectable in untreated epidermis, Snail mRNA levels were also normalized to Slug expression in untreated epidermis. The values obtained for Snail were multiplied by 1000 to allow them to be shown alongside values for Slug on this graph.

Enhanced Slug expression in response to UVR exposure of normal skin is transient; however, as skin tumors develop in response to chronic UVR exposure, Slug and Snail are persistently overexpressed [8]. To determine if Slug and Snail expression was also elevated in tumors induced by a two-stage DMBA/TPA protocol, we examined four DMBA/TPA-induced squamous cell carcinomas. All of these tumors had large numbers of cells with Slug and Snail-positive nuclei (Figure 1A). In well-differentiated tumors, Slug and Snail-positive nuclei were largely restricted to basal keratinocytes, while in less differentiated tumors, positive nuclei were more widely distributed. Both Slug and Snail proteins were localized to nuclei. Interestingly, we observed no cytoplasmic staining for Snail, although cytoplasmic localization has been reported by other investigators [25]. It is important to note that many of the available antibodies against Snail and Slug have poor specificity; however, the antibodies used for immunohistochemistry in our studies have been validated in both human and mouse tissues [26,27]. Increased expression of Slug and Snail proteins in skin tumors reflected increased expression of their mRNAs (Figure 1B). Expression of Slug mRNA was increased more than 20-fold in tumors compared to untreated epidermis. Snail mRNA was undetectable in untreated epidermis, but was evident in tumors. Although Snail was clearly expressed in skin tumors, Snail expression appeared to be lower than Slug expression and did not appear to be related to Slug expression.

### 3.2. TPA Induces Expression of Slug and Snail in Mouse Epidermis

**Figure 2:**
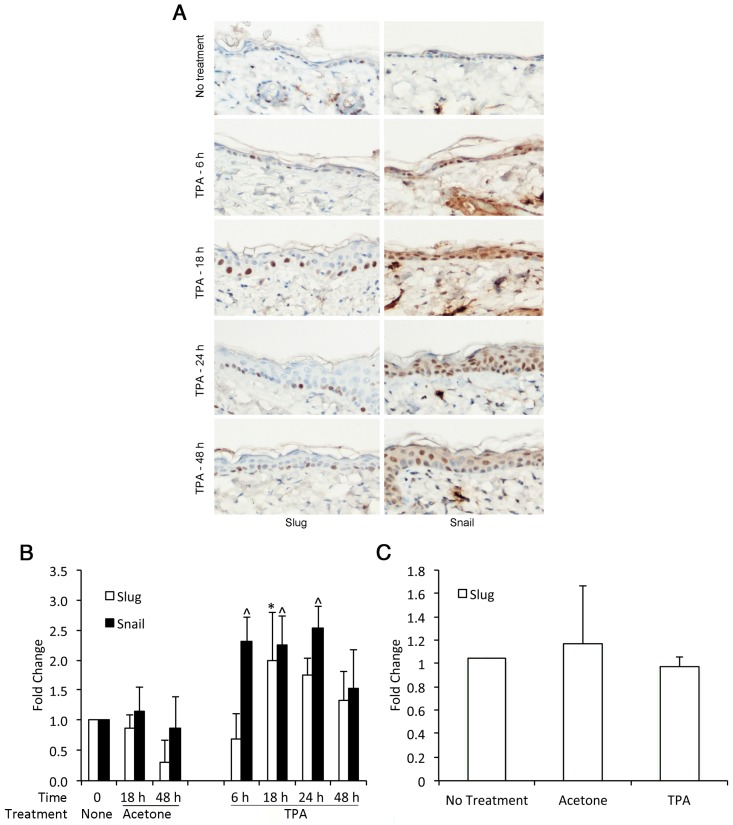
TPA induces Slug and Snail expression in the skin of mice. The shaved backs of 6–8-week-old female FVB mice were treated once with 2.5 µg TPA in acetone. Skin was harvested at different times after TPA application, and immunohistochemistry for Slug and Snail was performed. (**A**) Representative examples of immunohistochemical staining. (**B**) For quantification of Slug- and Snail-positive nuclei in TPA-treated skin, digitized slides were analyzed using the GENIE morphometry program, and the number of positive nuclei per mm was normalized to the number of positive nuclei in untreated skin. Results were compared by ANOVA followed by the Bonferroni correction. * Significantly elevated compared to 18 h and 48 h acetone-treated skin and 6 h TPA-treated skin. ^ Significantly different from 18 h and 48 h acetone-treated skin. Bars indicate SD. (**C**) Epidermal Slug and Snail mRNA levels at 18 h after TPA treatment were quantified. Snail mRNA was undetectable, while Slug levels were similar in untreated, acetone-treated and TPA-treated epidermis.

As previously reported, immunohistochemistry demonstrated small to moderate numbers of basal keratinocytes with Slug-positive nuclei in non-tumor epidermis [27] (Figure 2A). We also observed Snail-positive nuclei in unperturbed epidermis (Figure 2A), which has not been reported by others [28]. This finding suggested that the anti-Snail antibody used may have had some cross-reactivity with Slug. Acetone treatment alone did not significantly alter the number or distribution of Slug or Snail-positive nuclei. The number of Slug and Snail-positive nuclei in TPA-treated skin was increased at least two-fold compared to untreated or acetone-treated skin (Figure 2B). For Slug, TPA-induced expression appeared to be transient, with no increase in expression at 6 h, significantly increased expression at 18 h and declining expression thereafter. The kinetics of Snail induction were somewhat different. Snail expression was significantly increased by 6 h after TPA treatment and remained elevated for 24 h. We considered the possibility that the antibodies employed for immunohistochemistry lacked complete specificity for their target antigens. However, while Slug-positive nuclei were restricted to basal epithelium, Snail-positive nuclei were seen throughout the epidermis. Thus, it was unlikely that the monoclonal anti-Slug antibody was detecting Snail protein. It remained a possibility that a less specific polyclonal anti-Snail antibody was detecting both Slug and Snail. The different kinetics of Slug and Snail induction as revealed by immunohistochemistry, however, indicated that the anti-Snail and anti-Slug antibodies together were able to detect differences in Slug *versus* Snail induction kinetics. Increased expression of Slug protein was not reflected in increased mRNA expression at 18 h after TPA treatment (Figure 2C). Moreover, Snail mRNA was undetectable in untreated, acetone-treated or TPA-treated skin. It is likely that transient induction of Slug and Snail mRNA occurred earlier than the 18-h time point at which immunohistochemistry demonstrated expression of the proteins.

These responses were very similar to those seen after UVR exposure of the skin [7,27]. Expression of Slug protein as detected by immunohistochemistry is markedly elevated by 24 h after UVR exposure and remains elevated for at least 48 h. In UVR-exposed skin, induction of Slug and Snail mRNA is rapid, with maximal induction at 48 h post-exposure. Increased Snail mRNA expression is seen by 2 h post-exposure and increased Slug mRNA by 24 h. Thus, in both UVR and DMBA-/TPA-induced skin tumors, transient elevation of Slug and Snail expression in the epidermis becomes persistent as tumors develop.

### 3.3. Slug Modulates TPA-Induced Epidermal Hyperplasia

**Figure 3:**
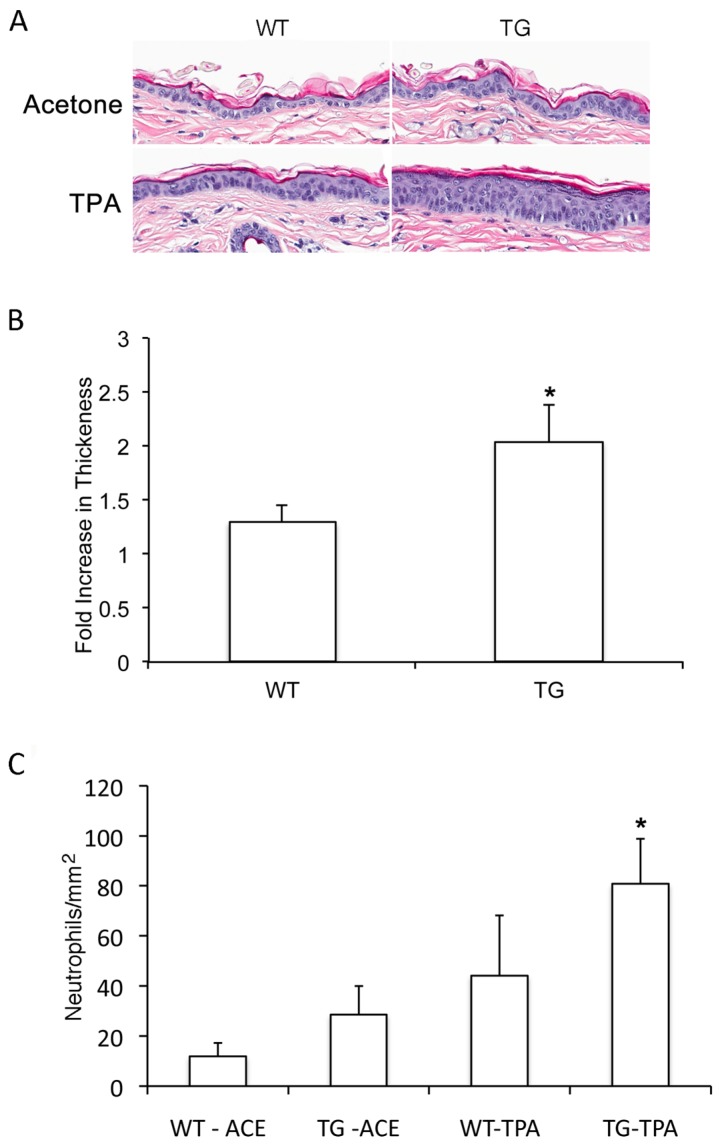
Slug modulates TPA-induced epidermal hyperplasia and acute cutaneous inflammation. Slug transgenic and wild-type mice were treated once with TPA, and skin was collected 24 h later. (**A**) H & E-staining showed enhanced hyperplasia in transgenic compared to wild-type epidermis treated with TPA. (**B**) Quantification of hyperplasia by morphometry showed a significant difference between the degree of hyperplasia in transgenic *versus* wild-type skin. Hyperplasia is shown as a fold increase in epidermal thickness compared to acetone-treated skin. Results were compared by the Mann–Whitney test. * One-tailed *p*-value significantly different from wild-type epidermis. Bars indicate SD. (**C**) Quantification of neutrophil number in skin was performed by staining for Ly6G followed by morphometry. Results were compared by the Mann–Whitney test. * One-tailed *p*-value significantly different from TPA-treated wild-type epidermis.

To determine if Slug expression was related to TPA-induced proliferation *in vivo*, we quantified TPA-induced epidermal hyperplasia in wild-type and Slug transgenic mice. Acetone-treated epidermis was slightly, but not significantly, increased in thickness compared to untreated epidermis. Acetone-treated wild-type and Slug transgenic epidermis did not differ in thickness, but Slug transgenic epidermis showed increased TPA-induced hyperplasia compared to wild-type epidermis (Figure 3). This indicates that Slug modulates, at least in part, hyperplasia in response to TPA.

### 3.4. Slug Plays a Role in TPA-Induced Acute Inflammation

Slug plays an important role in the acute inflammatory response to UVR, as shown by the fact that, compared to wild-type mice, Slug knockout mice show greatly reduced neutrophil influx into the skin after UVR exposure [10]. To determine if Slug also modulated the inflammatory response to TPA, we compared the response of Slug transgenic and wild-type mice. Compared to wild-type mice, Slug transgenic mice did not show significantly elevated neutrophil numbers in acetone-treated skin (Figure 3C); however, there was a significant, although modest increase in neutrophil influx in the transgenic *versus* wild-type skin after TPA treatment (Figure 3C). Thus, Slug influences the cutaneous inflammatory response of the skin to TPA, as well as to UVR.

### 3.5. COX-2 Expression is Unrelated to SLUG and Snail Induction by TPA

**Figure 4:**
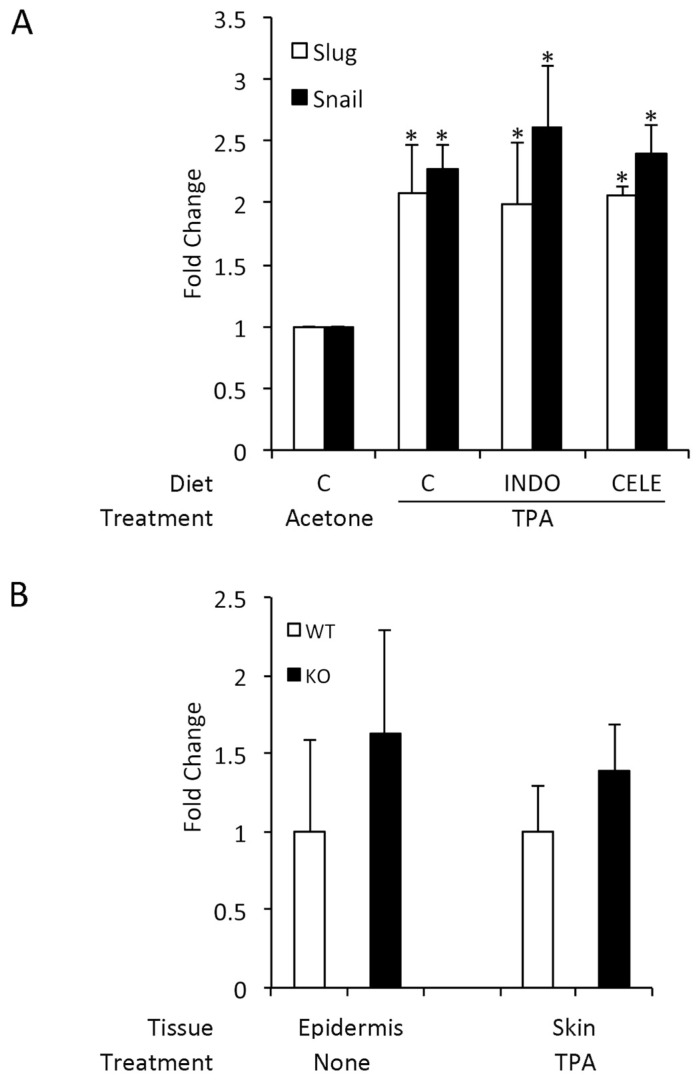
TPA induction of Slug and Snail induction are independent of COX-2 expression. (**A**) Slug and Snail induction in mice fed COX-2 inhibitors. Female FVB mice 6–8-weeks-old were fed a control diet (C) or one containing 4 ppm indomethacin (INDO) or 750 ppm celecoxib (CELE) for one week before application of 2.5 µg TPA in acetone to the shaved back. Eighteen hours after TPA application, mice were killed, and skin was removed for immunohistochemistry for Slug and Snail. The number of immunohistochemically-positive nuclei per mm was quantified morphometrically. Results are shown as the fold increase compared to acetone-treated skin of mice fed a control diet. Results were compared by ANOVA followed by the Bonferroni correction. * Significantly different from acetone-treated mice fed a control diet, but not different from TPA-treated mice fed any diet. Bars indicate SD. (**B**) PGE2 levels in TPA-treated wild-type and Slug knockout mice. PGE2 levels were determined in the epidermis of untreated wild-type and Slug knockout mice and in whole skin from TPA-treated wild-type and Slug knockout mice harvested 6 h after TPA treatment. In both cases, values in knockout samples were normalized to values in similarly treated wild-type samples. There was no significant difference between wild-type and knockout samples for either comparison when compared by the Mann–Whitney test. Bars indicate SEM.

Because there is a clear relationship between expression of COX-2, a potent mediator of cutaneous inflammation and EMT, perhaps mediated by Slug or Snail induction [15,16,17], and because TPA is a potent inducer of COX-2 [29,30], we investigated the role of COX-2 in modulating Slug and Snail expression. Animals were fed a control diet containing no additives, indomethacin (a general COX inhibitor) or celecoxib (a COX-2-selective inhibitor) for one week before the application of TPA. This treatment regime has been previously shown to inhibit prostaglandin production in the skin [30]. At 18 h post-treatment, the time of maximal Slug and Snail induction by TPA, there was no difference among the groups in Slug or Snail induction, as measured by the number of immunohistochemically-positive nuclei per mm of epidermis (Figure 4A). Thus, TPA did not appear to induce Snail family members via enhanced COX-2-dependent prostaglandin expression.

Another possible connection between Slug and COX-2 occurs via prostaglandin E2 (PGE2) catabolism, as Slug transcriptionally represses 15-hydroxyprostaglandin dehydrogenase, the enzyme that inactivates PGE2 [3,9,13,14]. Our studies failed to reveal a difference in PGE2 levels between the skin of wild-type and Slug knockout mice, whether untreated or TPA-treated (Figure 4B). Indeed, PGE2 levels in Slug knockout skin were slightly, although not significantly, higher than in wild-type skin.

The relationship between inflammation and expression of Snail family transcription factors is complex, and inflammatory mediators other than COX-2 are likely to be involved. The potent inflammatory cytokine tumor necrosis factor alpha (TNFα) has also been shown to induce expression of Slug and Snail in epithelial cells, and this induction is mediated via the NF-kappaB (NF-κB) pathway [31,32]. Thus, examination of this pathway would be valuable in future studies of Snail family factors during UVR and chemically-induced skin carcinogenesis.

### 3.6. TPA-Induced Changes in Slug and Snail Expression in Keratinocytes In Vitro Differ from In Vivo Findings

**Figure 5:**
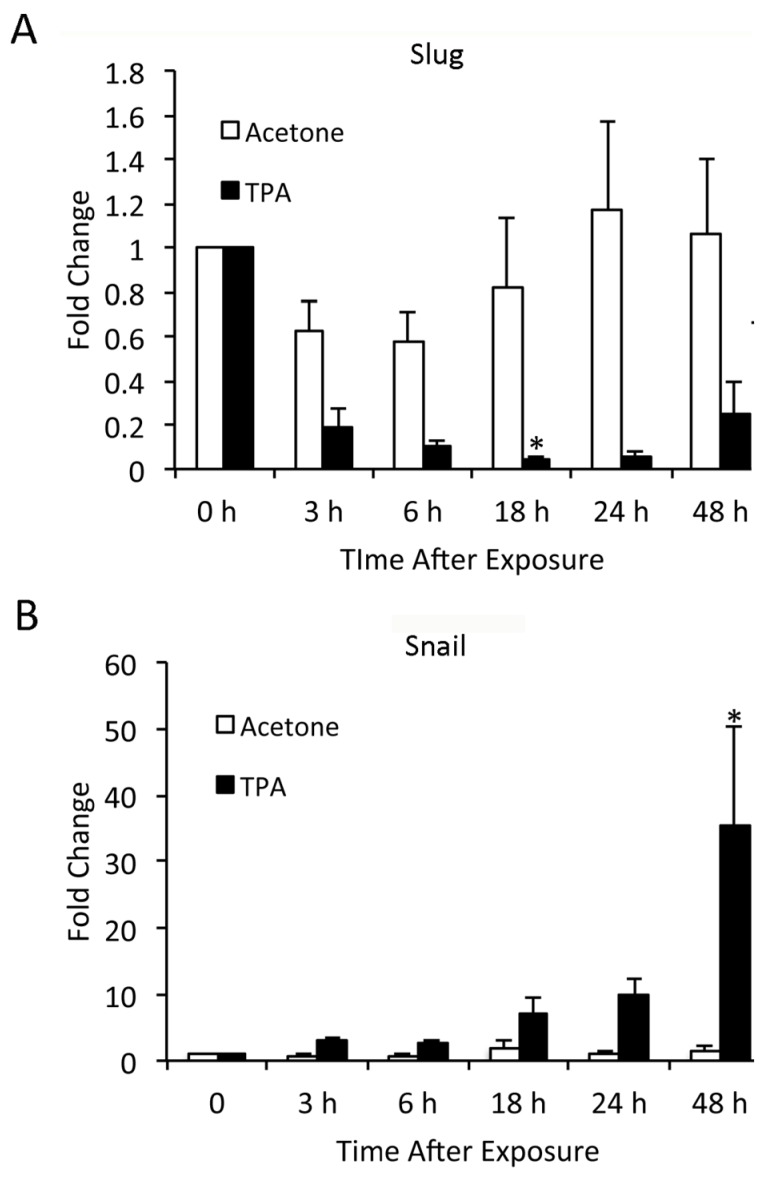

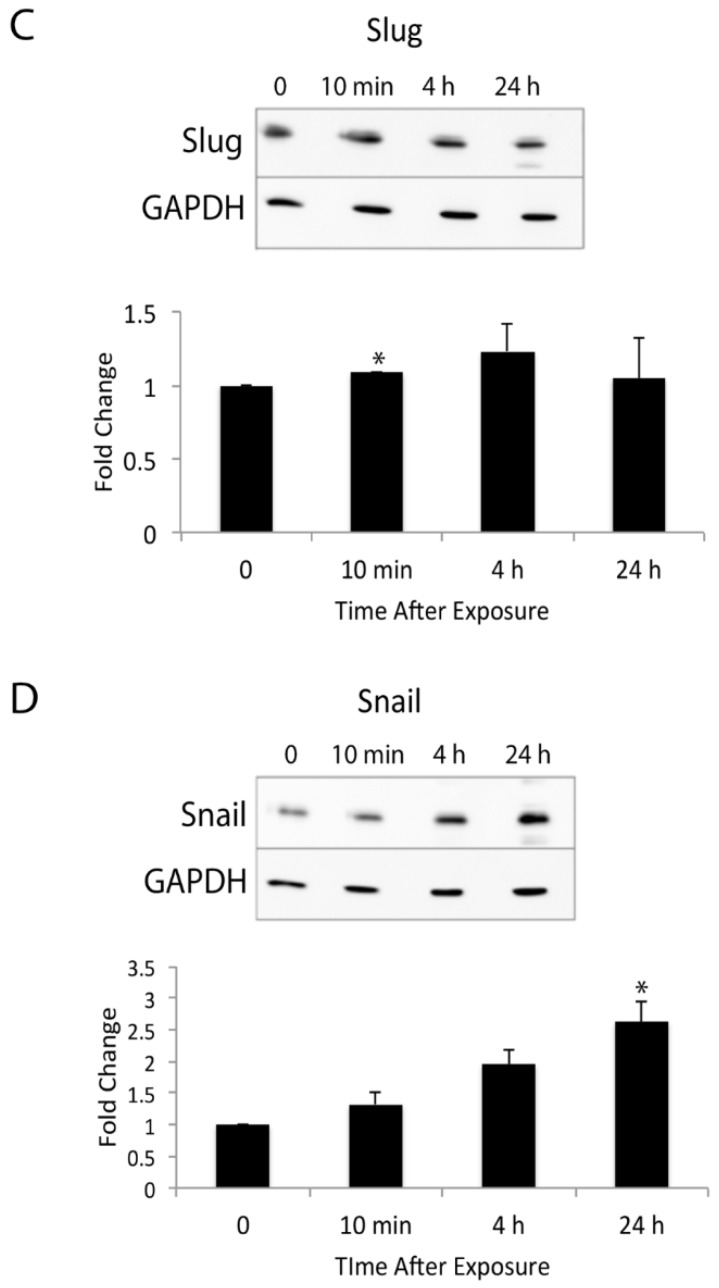
TPA alters the expression of Slug and Snail mRNA and protein in cultured NHK cells. (**A**,**B**) NHK cells were treated with TPA or acetone and harvested at later time points; 0 h samples were from untreated cells. mRNA was isolated, and Slug and Snail expression levels were determined by q-RT-PCR. Results are shown as a fold increase compared to 0 h samples. Results were compared by ANOVA followed by the Bonferroni correction. * Significantly different from 0 h samples. Bars indicate SEM. (**C**,**D**) SCC12F cells were treated with TPA and harvested at later time points; 0 h samples were from DMSO-treated cells. Protein was isolated, and Slug and Snail expression levels were determined by Western blotting followed by densitometry. Results are shown as a fold increase compared to DMSO-treated samples. Results were compared by a one-tailed Student *t*-test assuming unequal variance. * Significantly different from 0 h samples. Bars indicate SD.

In order to investigate in more detail the pathways by which TPA induced Slug and Snail expression in keratinocytes, we turned to *in vitro* studies of NHK. These studies revealed patterns of Slug and Snail induction quite different from those seen *in vivo* (Figure 5). TPA reduced Slug mRNA expression compared to untreated or acetone-treated cells for at least 48 h after its addition to the culture medium; this reduction was significant at 18 h following TPA treatment (Figure 5A). In contrast, TPA enhanced Snail expression; Snail expression continued to increase over time and was maximal at 48 h after TPA treatment (Figure 5B). Thus, expression of Slug and Snail *mRNA* in NHK was differentially regulated by TPA. When protein levels of Slug and Snail after TPA exposure were compared, changes in expression were much less marked than changes in mRNA (Figure 5C,D). Slug expression was minimally, but significantly increased at 10 min after TPA exposure and thereafter did not differ from the level in DMSO-treated cells (Figure 5C). In contrast, Snail protein expression progressively increased in TPA-treated samples and was elevated approximately 2.5-fold compared to DMSO-treated samples by 24 h after TPA application; this difference was statistically significant.

**Figure 6:**
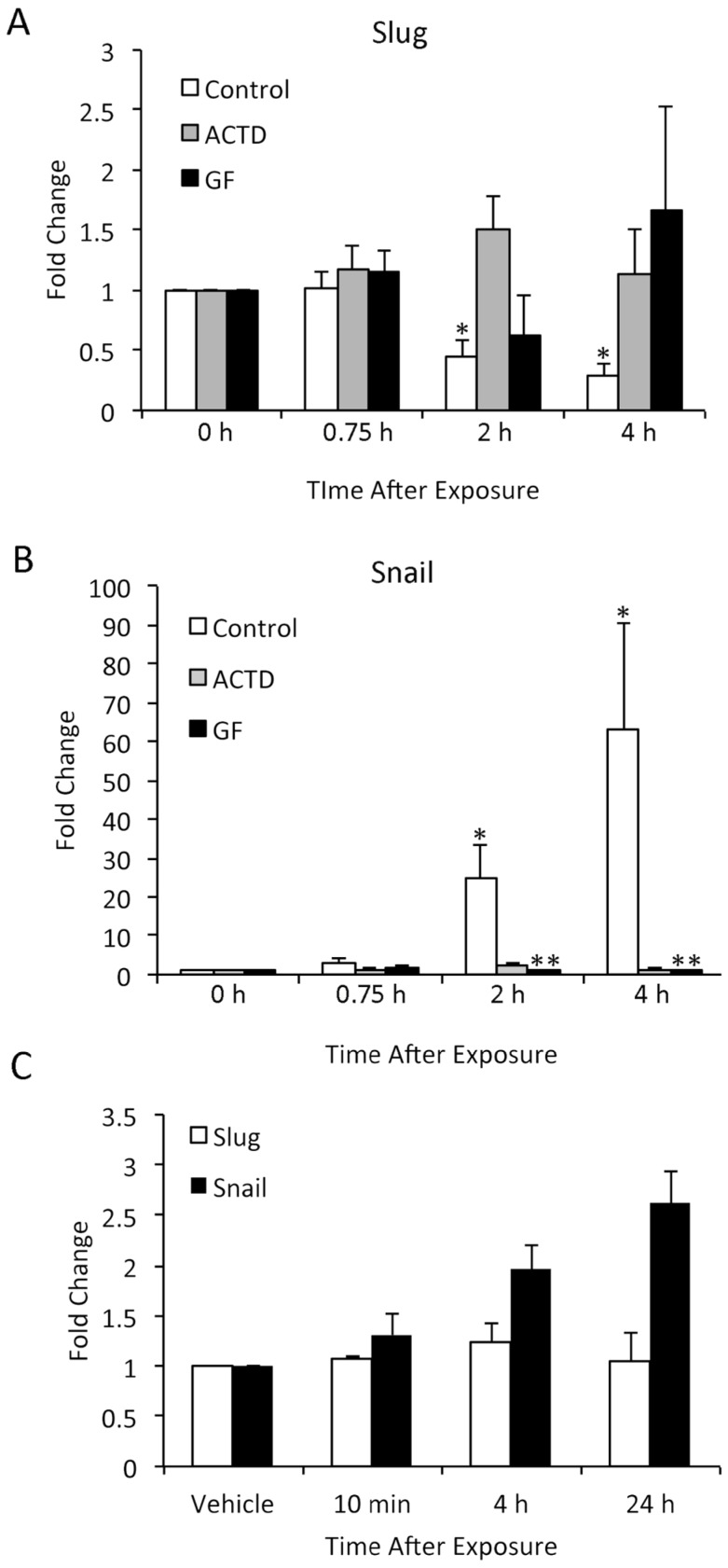
TPA alters the expression of Slug and Snail mRNA in cultured SCC12F cells, and inhibition of PKC activity or downstream signaling pathways abrogates this effect. (**A**,**B**) SCC 12F cells were treated with TPA in the presence of DMSO vehicle (**C**), actinomycin D (ACTD) or GF 109203X (GF). mRNA was isolated, and Slug (shown in 5A) and Snail (shown in 5B) expression levels were determined by q-RT-PCR. Results are shown as a fold increase compared to 0 h samples. Results were compared by the Kruskal–Wallis test followed by Dunn’s multiple comparison test. * Significantly different from the 0 h samples; ** significantly different from the control sample at that time point. Bars indicate SD. (**C**) Protein from vehicle or TPA-treated SCC 12F cells was analyzed by Western blotting for Slug and Snail, followed by densitometry. Values shown are the average of two independent replicates. Bars indicate the SD.

We performed similar studies in the SCC12F human epidermal cell line, using DMSO rather than acetone as a vehicle for TPA. SCC12F cells are transformed, but non-tumorigenic, and thus represent a model of early carcinogenesis. The response of SCC12F cells to TPA was very similar to that of NHK. At 2 and 4 h after TPA application, there was a significant decrease in Slug mRNA expression (Figure 6A) and a significant increase in Snail mRNA expression (Figure 6B). Actinomycin D, an inhibitor of RNA transcription, significantly inhibited TPA effects on Slug and Snail mRNA levels; thus, it appeared that mRNA synthesis was required for TPA activity. The effect of TPA on Slug and Snail mRNA levels could also be abrogated by blocking the TPA receptor PKC with the GF109203X inhibitor. This clearly showed that the effect of TPA on Slug and Snail mRNA levels was due specifically to activation of the TPA receptor and downstream signaling pathways, rather than to a more general cytotoxic effect of TPA or DMSO on cells. Interestingly, while TPA-induced increases in Snail RNA were reflected in increased protein levels, Slug protein levels in TPA-treated cells remained unchanged (Figure 6C).

The finding that the pattern of Slug induction by TPA *in vitro* was quite different from its induction *in vivo* was unexpected. It is unlikely that species differences were responsible for this dissimilarity, as Slug and Snail are similarly induced by acute UVR exposure in SCC12 human cells and mouse epidermis [7,8,27]. Moreover, Slug and Snail are persistently elevated both in human cutaneous squamous cell carcinomas and in UVR-induced skin tumors of mice [8,33]. This suggests that the regulation of their expression is similar in the two species. The difference between *in vivo* and *in vitro* responses may be explained by the previous observation that TPA induces keratinocyte proliferation *in vivo*, but keratinocyte differentiation *in vitro* [34]. Since Slug is ordinarily expressed only in basal keratinocytes, the differentiation effects of TPA may have resulted in Slug downregulation *in vitro*. On the other hand, Snail is not ordinarily expressed in the epidermis; thus, it may play little or no role in keratinocyte differentiation and may have been responsive to TPA induction *in vitro*.

Another unexpected finding was that TPA induction of Snail mRNA was reflected in Snail protein levels in TPA-treated cells, but TPA-induced reduction in Slug mRNA expression was not associated with decreased Slug protein levels. This difference may be due to differential protein stability, as both Snail and Slug are unstable proteins with half-lives of less than an hour due to ubiquitin-mediated degradation [35,36]. However, the means by which they are targeted for ubiquitination and the factors that modulate targeting and ubiquitination differ. While Snail is degraded primarily via GSK-3β-dependent phosphorylation and β-TrCP-mediated ubiquitination, Slug is degraded via p53-driven, MDM2-mediated ubiquitination.

## 4. Conclusions

In the present studies, we investigated the role the Slug transcription factor plays in chemically-induced skin cancer. Expression of Slug and the related transcription factor Snail was elevated in skin tumors produced in response to a two-stage protocol, using DMBA as the initiator and TPA as the promoter. TPA rapidly induced transient elevation of Slug and Snail mRNA and protein in uninitiated skin; their induction appeared to be independent of COX-2 levels. Slug modulated, at least in part, TPA-induced epidermal hyperplasia and skin inflammation. These studies indicate that Slug and Snail exhibit similar patterns of expression and likely play similar roles in both UVR and DMBA-/TPA-induced squamous cell carcinomas of mice. *In vitro* studies revealed patterns of Snail and Slug expression in response to TPA that differed markedly from *in vivo* findings. In cultured human keratinocytes, TPA enhanced Snail expression, but suppressed Slug expression. Thus, *in vitro* studies of Snail family factors may not accurately recapitulate *in vivo* findings, and under some conditions, Slug and Snail may be differentially regulated by extrinsic factors.
